# Sociodemographic Correlates of Unipolar Major Depression among the Chinese Elderly in Klang Valley, Malaysia: An Epidemiological Study

**DOI:** 10.1155/2014/812712

**Published:** 2014-12-02

**Authors:** Rohit Kumar Verma, Tan Hui Min, Srikumar Chakravarthy, Ankur Barua, Nilamadhab Kar

**Affiliations:** ^1^Department of Pharmacy Practice, School of Pharmacy, International Medical University, Bukit Jalil, 57000 Kuala Lumpur, Malaysia; ^2^Department of Pathology, School of Medicine, International Medical University, Bukit Jalil, 57000 Kuala Lumpur, Malaysia; ^3^Department of Community Medicine, School of Medicine, International Medical University, Bukit Jalil, 57000 Kuala Lumpur, Malaysia; ^4^Department of Psychiatry, Black Country Partnership NHS Foundation Trust, Wolverhampton WV10 9TH, UK

## Abstract

*Background*. Depression, as one of the most disabling diseases around the world, had caught the global concern with its rising prevalence rate. There is a growing need of detecting depression, particularly in the old age population which is often left being overlooked. *Methods*. We conducted a cross-sectional community-based study which included 150 Chinese elderly aged 60 and above within Klang Valley area. We obtained the sociodemographic profiles and assessed the status of well-being, depression, and cognitive function of the participants with the help of instruments: WHO Five-Item Well-Being Index, Major (ICD-10) Depression Inventory, and 6-Item Cognitive Impairment Test. *Results*. We found that the prevalence of depression among the Chinese elderly within Klang Valley region was 10.7%. With multiple logistic regression, decision to consult doctor on depressed mood or memory problem and presence of cognitive impairment were shown to be significantly associated with unipolar major depression, whereas wellbeing status was also found to be statistically correlated with depression in univariate analysis. *Conclusion*. The prevalence of unipolar depression among Chinese elderly within Klang Valley, Malaysia presented that there was an increased trend compared to the previous studies.

## 1. Introduction 

Depression, particularly unipolar major depression, is characterized by sadness, loss of interest in daily activities, negative self-regard, troubled sleeping or change in appetite, fatigue, and poor concentration, which is adapted from World Health Organization [1]. Depressive disorders can be unipolar or bipolar, with unipolar major depression as the more prevalent form than bipolar depressive state. Bipolar mood disorder has both mania state and depressed state in the course of the illness, while the absence of mania state in unipolar major depression is the key to differentiate them [2].

Being one of the most common disorders of all psychiatric disorders in geriatric population, it also appeared as one of the leading causes of global disease burden following ischemic heart disease [3]. In Malaysia, major depression is one of the top three leading causes of disability, according to Global Burden of Disease Profile Malaysia in year 2010 [4]. Studies in other regions had found that geriatric depression is associated with sociodemographic factors and present physical illnesses [5–8].

To date, there are only a scarce number of studies for geriatric depression in general community setting carried out within Klang Valley region. The most recent study on geriatric depression among the community was done 8 years ago (year 2005) [9]. As the population is ageing due to increase in life expectancy, the proportion of elderly in the incidence of depression may increase. If depression in elderly is left undiagnosed and untreated, it could result in reduced quality of later life and probably worsening in health conditions, which eventually leads to increase in morbidity, mortality, and health care costs.

### 1.1. Objectives

This community-based study was aimed at determining the prevalence of unipolar major depression among the Chinese elderly in Klang Valley, Malaysia. Another objective was to study the sociodemographic and clinical variables associated with the depression in this population.

## 2. Materials and Methods

### 2.1. Study Design and Setting

It is a cross-sectional, epidemiological study which was conducted from April to November 2013 in Klang Valley region, Malaysia. Selected study area is the main economic and cultural core of Malaysia, as well as the most densely populated region in Malaysia, which consists of approximately 6 million people. Klang Valley comprises Wilayah Persekutuan Kuala Lumpur, Wilayah Putrajaya, and subdistricts of Selangor state (Gombak, Hulu Langat, Sepang, Petaling, and Klang).

### 2.2. Sample Size Estimation

The sample size was estimated by using the sample size formula for finite population:

(1) $$\begin{array}{c} \frac{Nt^{2}pq}{d^{2}\left(N-1\right)+t^{2}pq}. \end{array}$$

Here, the confidence interval (CI) was taken as 95%.

*t* is normal deviate corresponding to the required CI.

Here, it was** 1.96** for 95% CI.

*p* is** 6.3**% prevalence rate of depression in elderly in Malaysia = 0.063;

*q* is (1 − *p*). Here it is (1 − 0.063) =** 0.937**.

*d* is allowable error; 5% = 0.05.

*N* is total geriatric population (≥60 yrs.) within Klang Valley regions =** 395276**.

The total geriatric population aged 60 and above within the Klang Valley was estimated by addition of the number of citizens (≥60 years) in all the districts within Klang Valley region taken from the population distribution statistics in year 2010 which is available on the official portal of Department of Statistics Malaysia [10]. The prevalence rate of depression in elderly in Malaysia was set as 6.3%, taken from a study of prevalence of depression in elderly in Bandar Baru Bangi, Malaysia, in year 2005 [11]. The sample size was estimated to be 91 and cross-checked with RAOSOFT calculator, an online tool used for sample size estimation. We included additional 10% (9 subjects) to the sample size to provide for the nonresponse rate, therefore making the final minimum required sample size as 100.

### 2.3. Study Participants

Eligibility criteria included that the respondent must be a Chinese aged 60 years or more and staying within Klang Valley area. We excluded subjects who were unable to participate in the study or provide a consent (either verbal or written) for any cause, which included terminal illness and severe auditory or articulation impairment, and if the interview was terminated prematurely and could not be continued in later time. Interviews were mainly conducted in Mandarin Chinese, English, and some other dialects such as Cantonese and Hokkien. The subjects would be excluded if there is a communication barrier between investigator and the subject.

### 2.4. Study Samples and Recruitment

During the data collection, we applied chain referral sampling, namely, Snowball sampling which is a nonprobability sampling technique. It was useful for the study in which the potential subjects are difficult to locate. First, we identified a house with an elderly who can be a potential respondent. Following his/her participation in the study, this elderly was asked to locate other nearby households containing potential subjects at the end of the interview. This process was repeated until sufficient samples were obtained.

For data collection the subject was initially screened for the eligibility criteria and given information about the study. If eligible and the subject consented to participate we presented the participant with either the English or the Chinese version of the study information sheet according to the participant's language preference. The name and identification card number were not recorded in the response sheet to assure the confidentiality and anonymity of the respondent. Instead, all the respondents were coded with a series of numbers.

### 2.5. Questionnaire

A detailed questionnaire including sociodemographic characteristics, financial dependency, and chronic comorbid conditions was utilized. This questionnaire was also attached with instruments including WHO Five-Item (WHO-5) Well-Being index, Major (ICD-10) Depression Inventory (MDI), and 6-Item Cognitive Impairment Test (6CIT) to assess the subjects' well-being status, presence of unipolar major depression, and cognitive impairment. These instruments were adapted from World Health Organization, which are well established.

We had translated the questionnaire and instruments into Mandarin Chinese to ease the understanding for those subjects who cannot read English language. Pilot study was conducted on 35 Chinese elderly to evaluate the reliability of the translated questionnaire before its execution in the actual study.

### 2.6. Study Variables

We obtained data including household information, financial status, and health conditions via direct questioning during the interview. Presence of chronic illness was determined according to subjects' self-reported illnesses that were diagnosed, were under treatment, or were followed up by doctors at medical facilities. We also assessed their well-being status, presence of depressive symptoms, and cognitive function with the help of the following instruments.

WHO-5 Well-Being Index was used to assess the subjective quality of life in the participant. The questions were asked to examine the level of positive mood (good spirits and relaxation), vitality (active life and good rested), and general interest in daily life. Any of the items gives a score of 0 or a total score of less than 13, indicating poor well-being and high tendency of being depressed.

Major (ICD-10) Depression Inventory was used to validate the depression status in those who obtain an unoptimistic result in the well-being status test. Scoring 4 or 5 (including score of 3 for the last 5 items) is indicative of the presence of clinical depression. Based on different combination scoring, we classified the participant into nondepressed, mildly, moderately, or severely depressed.

Presence of cognitive impairment was assessed with the use of 6CIT Dementia Test, a simple test with high specificity and sensitivity A score of more than 7 (>= 8) indicates presence of cognitive impairment. Particularly, score of 8 to 9 represents mild cognitive impairment while a score of 10 or more means severe cognitive impairment.

### 2.7. Ethical Approval and Consent

This research was reviewed and approved by International Medical University Joint-Committee of the Research and the Ethics Committee (Ethical Approval number: B. Pharm B01/10-Res (37) 2013). All the subjects participating in the study were required to provide informed consent either verbally or in written form prior to the interview.

### 2.8. Statistical Analysis

We entered and analyzed the data using Statistical Package for Social Science (SPSS) version 22.0, with a 95% confidence interval (CI) and a significance level of 0.05. To determine the correlation between the variables and depression, we applied Pearson's Chi-square test, whereas we used Fisher's exact test instead of Pearson's Chi-square test for the variables with expected count of less than five.

#### 2.8.1. Descriptive Statistics

Descriptive statistics were used to determine the prevalence of unipolar major depression in elderly. It provided the baseline frequencies of the study population.

#### 2.8.2. Univariate Analysis

Univariate analysis was used to study the relationship between the sociodemographic variables and other variables with depression status. The covariates were regressed individually with depression status to obtain a rough estimate of odds ratio (OR) and the CIs. Among the covariates, total score of WHO-5 Well-Being Index and total score of 6CIT Dementia Test were continuous variables, while the rest were categorical variables.

#### 2.8.3. Multivariate Analysis

Multiple logistic regression (MLR) was used to study the effect of more than one independent variable on the one dichotomous outcome (dependent variable: depression status). Covariates with *P* value of < 0.30 in univariate analysis were included in the multivariate analysis for adjustment. The final multivariable model included significant variables with *P* < 0.05.

Receiver operating curve (ROC) presents the tradeoff point between specificity and sensitivity. The area under the curve represents the accuracy of the instrument, in which the disease group can be being well separated from those without disease by using the instruments mentioned. Area under curve of 0.80 and above shows that the instruments possess a good level of accuracy.

## 3. Results

A total of 159 subjects agreed to participate in the interview. We excluded 9 individuals due to premature termination or because they were staying at places that were out of the field of research. Total number of elderly contacted was not recorded, including those who refused and those who were unable to respond properly due to severe cognitive, auditory, or articulation impairment. Therefore, we had only taken the data of 150 subjects for statistical analysis.

The baseline characteristics of the subjects interviewed showed that 49.3% were males and 50.7% were females. Around half of the respondents (51.3%) aged from 60 to 69 years, while 9.3% of them were found to be cognitively impaired (mild and severe).

Table 1 shows that 58.0% of individuals belonged to Kuala Lumpur (KL) area, while the remaining came from outskirts of KL. The majority (68.7%) were married, while the rest were either single or widowed. Only 8.0% were staying alone. The majority of them were retired (84.0%), literate (50.7%), nonsmokers (79.3%), nonalcoholic (90.0%), and financially dependent (61.3%). Around half (54.7%) of the respondents reported having 3 or more chronic diseases, and most of respondents (91.3%) were under treatment and were followed up.

Table 2 presents the association between depression and each of the variables according to univariate analysis. The correlation between depression and cognitive impairment was found to be statistically significant (*χ* ^2^ = 10.166, df = 1, *P* = 0.008). There is also statistically significant association between depression and the decision to consult a doctor for depressed mood and memory problem (*χ* ^2^ = 23.985, df = 1, *P* = 0.000). Well-being status based on the scoring in WHO-5 Well-being Index was also found to be statistically correlated with depression (*χ* ^2^ = 123.488, df = 1, *P* = 0.000), but the odds ratio could not be determined due to one of the cells (25%) having expected count of less than five (listed as confounder and excluded in MLR model).

Table 3 shows the multivariate correlation between unipolar major depression and the variables. In this multivariate analysis, decision to consult doctor on depressed mood or memory problem (“No”: adjusted OR = 0.074, 95% CI = 0.016–0.346) and presence of cognitive impairment (“Present”: adjusted OR = 8.115, 95% CI = 1.670–39.441) were discovered as the significant predictors of unipolar major depression.

**Figure 1:**
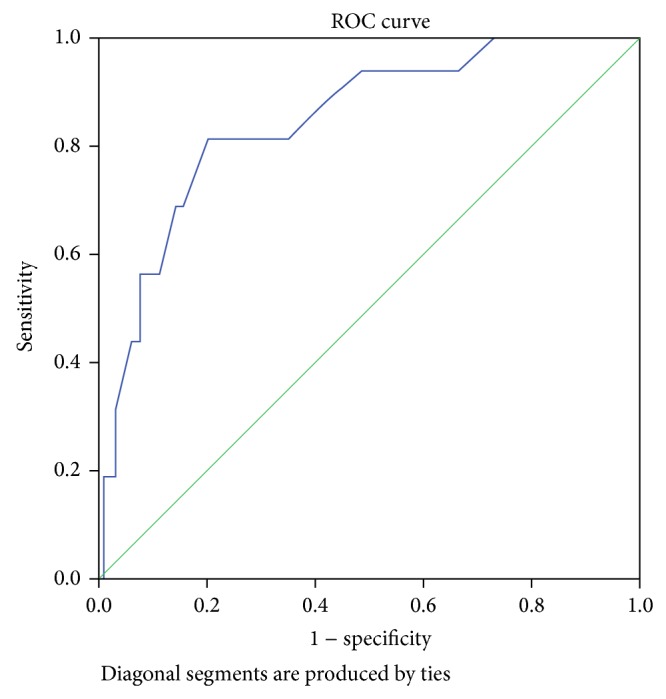
ROC curve: assessment of external validity of instruments. This graph was produced by SPSS 22.0 software.

Figure 1 illustrates the results of ROC curve for assessment of external validity and accuracy of WHO-5 Well-Being Index (1998 version), Major (ICD-10) Depression Inventory, and Six-Item Cognitive Impairment Test. The total area under the curve was 0.846, displaying the fact that these instruments possess a good accuracy.

## 4. Discussion

### 4.1. Prevalence of Unipolar Major Depression

Among the study of prevalence of geriatric depression in Malaysia, this is the pioneer study that used WHO validated questionnaire and instruments to determine the prevalence of unipolar major depression among Chinese elderly population within Klang Valley region, Malaysia [9].

In our study, the prevalence of depression among the Chinese elderly was found to be 10.7% (95% CI = 5.7–15.6). This result was cohering the WHO's projection on the overall prevalence rate of geriatric depression, which is 10% to 20%, depending on cultural differences [12]. The most recent community-based study in year 2005 revealed that the prevalence of geriatric depression regardless of races within an urban area in Malaysia was 6.3% [9]. The observed difference reflects the increasing trend in the rate of geriatric depression in Malaysia, which indicates the need for focused assessment to identify depression in elderly, to study the contributing factors, and to develop appropriate interventions on late life depression.

### 4.2. Sociodemographic and Health Related Correlates

Our study found that the presence of cognitive impairment, poor well-being, and the decision to acquire doctor's consultation for depressed mood or memory problem (No) were the significant predictors of unipolar major depression.

In our study, those who presented with impaired cognitive function were 8.1 times more likely to be associated with depression. Association of depression with cognitive impairment in elderly has been reported. A study in the Netherland on 500 subjects of age 85 years revealed that an accelerated increase of depressive symptoms annually was highly associated with some of the symptoms of cognitive impairment (i.e., impaired attention and delayed recall) at baseline, while depressive symptoms were not associated with an accelerated decrease in cognitive function [13]. Meanwhile, in a voxel-based morphometric study on 72 participants, late life depression was associated with an increased risk of Alzheimer's disease incidence in which there was observable diminished gray matter volume [14]. Another study reported that patients who suffered more severe cognitive impairment endorsed a greater level of social withdrawal and lesser psychomotor agitation, which is independent of their underlying depression severity [15]. A follow-up study of one-year duration found that older adults suffered acute depression; the mild cognitive impairment occurring during the depressed state persisted even after depression was remitted [16]. These studies indicate the possibility of a bidirectional association between depression and cognitive impairment, and this suggests a clinical implication for the exploration of comorbidity when one of the two disorders is present in an elderly and to appropriately address the intervention approach.

For those who decided to consult or have consulted a doctor, the degree of depressive symptoms had reached a certain level that can severely affect the daily living of the individual, whereas, for those who do not think of consulting a doctor, the depressive symptoms were likely to be mild and the subjects often are unaware of them. The relationship between depression and the decision to seek doctor's consultation on depressed mood could be causal as supported by another study [17]; however this was contributed by depression severity and effect on functioning.

In univariate analysis, we found that well-being status possessed a very significant association with depression. However, our study could not show the nature of the relationship because the odds ratio was unable to be determined as mentioned above (refer to Section 3). It has been reported in a study in India that depression was much more prevalent in those who scored poorly in WHO-5 Well-Being Index, compared to those having satisfactory well-being status [18].

### 4.3. Limitations

While the study sample could suggest the prevalence of depression in Chinese elderly, inadequate sample in different subgroups of variables and sample with contributing factors were small. A larger sample size may be helpful to confirm the contributing factors. Household surveys resulted in unfeasible access to those who were homeless, living in care centers or in hospitals. Elderly who were excluded due to failure in meeting the criteria or due to communication barrier were unable to be evaluated, resulting in undervalued prevalence rate. In addition, bias may occur during process of self-reporting the chronic illnesses and other sociodemographic information and during the assessment of well-being and depression status.

## 5. Conclusion

In this study, prevalence of unipolar major depression among the Chinese elderly within Klang Valley was 10.7%. Presence of cognitive impairment, well-being status, and decision to acquire doctor's consultation on depressed mood or memory problem were found to be significantly associated with unipolar major depression. Study findings are helpful to provide the decision making approach for policy makers dealing with geriatric care to provide value in health.

## Figures and Tables

**Table 1:** Sociodemographic profile of the elderly Chinese in Klang Valley, Malaysia (categorical variables).

Baseline characteristics (categorical variables)	Depression		
	Present *N*_1_ (%)	Absent *N*_2_ (%)	Total surveyed *N* (%)
Age			
80 y/o and above	2 (1.3)	5 (3.3)	7 (4.7)
70–79 y/o	8 (5.3)	58 (38.7)	66 (44.0)
60–69 y/o	6 (4.0)	71 (47.3)	77 (51.3)
Gender			
Male	7 (4.7)	67 (44.7)	74 (49.3)
Female	9 (6.0)	67 (44.7)	76 (50.7)
Area			
KL	9 (6.0)	78 (52.0)	87 (58.0)
Outskirt of KL	7 (4.7)	56 (37.3)	63 (42.0)
Type of family			
Nuclear	9 (7.6)	60 (50.8)	69 (58.5)
Joint/extended	4 (3.4)	45 (38.1)	49 (41.5)
Staying with			
Alone	1 (0.7)	11 (7.3)	12 (8.0)
With family	15 (10.0)	123 (82.0)	138 (92.0)
Marital status			
Single/widowed/separated/divorced	5 (3.3)	42 (28.0)	47 (31.3)
Married	11 (7.3)	92 (61.3)	103 (68.7)
Previous occupation			
Unemployed	1 (0.7)	18 (12.0)	19 (12.7)
Unskilled	8 (5.3)	38 (25.3)	46 (30.7)
Skilled	6 (4.0)	62 (41.3)	68 (45.3)
Professional	1 (0.7)	16 (10.7)	17 (11.3)
Present occupation			
Unemployed	2 (1.3)	19 (12.7)	21 (14.0)
Retired	13 (8.7)	92 (61.3)	105 (70.0)
Unskilled	1 (0.7)	9 (6.0)	10 (6.7)
Skilled	0 (0.0)	11 (7.3)	11 (7.3)
Professional	0 (0.0)	3 (2.0)	3 (2.0)
Literacy			
Illiterate	10 (6.7)	64 (42.7)	74 (49.3)
Literate	6 (4.0)	70 (46.7)	76 (50.7)
Smoking			
Smoking	3 (2.0)	28 (18.7)	31 (20.7)
Nonsmoking	13 (8.7)	106 (70.7)	119 (79.3)
Alcohol consumption			
Alcoholic	1 (0.7)	14 (9.3)	15 (10.0)
Nonalcoholic	15 (10.0)	120 (80.0)	135 (90.0)
Presence of comorbidity			
Present	16 (10.7)	127 (84.7)	143 (95.3)
Absent	0 (0.0)	7 (4.7)	7 (4.7)
Disease category (by number of disease)			
3 diseases and above are present	12 (8.0)	70 (46.7)	82 (54.7)
Less than 3 diseases are present	4 (2.7)	64 (42.7)	68 (45.3)
Consult doctor on health problem?			
No	2 (1.3)	11 (7.3)	13 (8.7)
Yes	14 (9.3)	123 (82.0)	137 (91.3)
Consult doctor on depressed mood/memory problem?			
No	10 (6.7)	129 (86.0)	139 (92.7)
Yes	6 (4.0)	5 (3.3)	11 (7.3)
Family history of			
(a) Psychiatric illness			
Yes	3 (2.0)	9 (6.0)	12 (8.0)
No	13 (8.7)	125 (83.3)	138 (92.0)
(b) Suicide attempt			
Yes	1 (0.7)	0 (0.0)	1 (0.7)
No	15 (93.8)	134 (89.3)	149 (99.3)
Types of psychiatric event in family history			
Depression	2 (1.3)	5 (3.3)	7 (4.7)
Schizophrenia	1 (0.7)	1 (0.7)	2 (1.3)
Alzheimer's disease	0 (0.0)	3 (2.0)	3 (2.0)
Financial dependency			
Totally dependent	10 (6.7)	48 (32.0)	58 (38.7)
Partially dependent	0 (0.0)	22 (14.7)	22 (14.7)
Independent	6 (4.0)	52 (34.7)	58 (38.7)
Wellbeing status			
Poor	16 (10.7)	3 (2.0)	19 (12.7)
Satisfactory	0 (0.0)	131 (87.3)	131 (87.3)
Cognitive impairment			
Present	5 (3.3)	9 (6.0)	14 (9.3)
Absent	11 (7.3)	125 (83.3)	136 (90.7)
Severity of cognitive impairment			
Significant	4 (2.7)	5 (3.3)	9 (6.0)
Mild	1 (0.7)	4 (2.7)	5 (3.3)
None	11 (7.3)	125 (83.3)	136 (90.7)

**Table 2:** Univariate analysis: sociodemographic correlates of depression.

Sociodemographic correlates (categorical variables)	Depression			*P* value	OR (unadjusted)	95% CI
	Present *N*_1_ (%)	Absent *N*_2_ (%)	Total surveyed *N* (%)			
Age group						
70 y/o and above	10 (6.7)	63 (42.0)	73 (48.7)	**0.241**	1.878	0.646–5.462
60–69 y/o	6 (4.0)	71 (47.3)	77 (51.3)			
Gender						
Male	7 (4.7)	67 (44.7)	74 (49.3)	0.636	0.778	0.274–2.210
Female	9 (6.0)	67 (44.7)	76 (50.7)			
Area						
KL	9 (6.0)	78 (52.0)	87 (58.0)	0.881	0.923	0.324–2.626
Outskirt of KL	7 (4.7)	56 (37.3)	63 (42.0)			
Type of family						
Nuclear	9 (7.6)	60 (50.8)	69 (58.5)	0.404	1.688	0.489–5.829
Joint/extended	4 (3.4)	45 (38.1)	49 (41.5)			
Staying with						
Alone	1 (0.7)	11 (7.3)	12 (8.0)	0.626^*^	0.745	0.090–6.187
With family	15 (10.0)	123 (82.0)	138 (92.0)			
Marital status						
Single/widowed/separated/divorced	5 (3.3)	42 (28.0)	47 (31.3)	0.994	0.996	0.325–3.047
Married	11 (7.3)	92 (61.3)	103 (68.7)			
Previous occupation						
Unemployed	1 (0.7)	18 (12.0)	19 (12.7)	0.695^*^	0.430	0.064–3.284
Employed	15 (10.0)	116 (77.3)	131 (87.3)			
Present occupation						
Unemployed	15 (10.0)	111 (74.0)	126 (84.0)	0.470^*^	3.108	0.391–24.716
Employed	1 (0.7)	23 (15.3)	24 (16.0)			
Literacy						
Illiterate	10 (6.7)	64 (42.7)	74 (49.3)	**0.265**	1.823	0.627–5.301
Literate	6 (4.0)	70 (46.7)	76 (50.7)			
Smoking						
Regular smoker/ex-smoker	3 (2.0)	28 (18.7)	31 (20.7)	0.570^*^	0.874	0.233–3.279
Nonsmoker/occasional	13 (8.7)	106 (70.7)	119 (79.3)			
Alcohol consumption						
Current or past alcohol abuse/dependence	1 (0.7)	14 (9.3)	15 (10.0)	0.505^*^	0.571	0.070–4.660
No alcohol use/occasional	15 (10.0)	120 (80.0)	135 (90.0)			
Disease category (by number of disease)						
3 diseases and above are present	12 (8.0)	70 (46.7)	82 (54.7)	**0.084**	2.743	0.842–8.938
Less than 3 diseases are present	4 (2.7)	64 (42.7)	68 (45.3)			
Consult doctor on health problem?						
No	2 (1.3)	11 (7.3)	13 (8.7)	0.632^*^	1.597	0.321–7.951
Yes	14 (9.3)	123 (82.0)	137 (91.3)			
Consult doctor on depressed mood/memory problem?						
No	10 (6.7)	129 (86.0)	139 (92.7)	**0.000** ^*^	0.065	0.017–0.249
Yes	6 (4.0)	5 (3.3)	11 (7.3)			
Family history of						
(a) Psychiatric illness						
Yes	3 (2.0)	9 (6.0)	12 (8.0)	**0.120** ^*^	3.205	0.770–13.340
No	13 (8.7)	125 (83.3)	138 (92.0)			
(b) Suicide attempt						
Yes	1 (0.7)	0 (0.0)	1 (0.7)	**0.107** ^*^	9.933	6.147–16.052
No	15 (93.8)	134 (89.3)	149 (99.3)			
Financial dependency						
Dependent	10 (6.7)	82 (54.7)	92 (61.3)	0.919	1.057	0.362–3.082
Independent	6 (4.0)	52 (34.7)	58 (38.7)			
Well-being status						
Poor	16 (10.7)	3 (2.0)	19 (12.7)	**0.000** ^*^	—	—
Satisfactory	0 (0.0)	131 (87.3)	131 (87.3)			
Cognitive impairment						
Present	5 (3.3)	9 (6.0)	14 (9.3)	**0.008** ^*^	6.313	1.800–22.146
Absent	11 (7.3)	125 (83.3)	136 (90.7)			

^*^ *P* value was produced with Fisher's exact test; bolded figures (*P* value < 0.30) showed that the corresponding variables were included in the multivariate analysis.

**Table 3:** Multivariate analysis: sociodemographic correlates of depression.

Sociodemographic correlates (categorical variables)	OR (adjusted)	*P* value	95% CI
Age group			
70 y/o and above	0.940	0.933	0.223–3.967
60–69 y/o	1.000		
Literacy			
Illiterate	1.029	0.967	0.262–4.047
Literate	1.000		
Disease category (by number of disease)			
3 diseases and above are present	2.676	0.181	0.633–11.308
Less than 3 diseases are present	1.000		
Consult doctor for depressed/memory problem?			
No	0.074	**0.001**	0.016–0.346
Yes	1.000		
Family history of psychiatric illness			
Yes	2.883	0.255	0.466–17.840
No	1.000		
Cognitive impairment			
Present	8.115	**0.009**	1.670–39.441
Absent	1.000		

Bolded figures showed that the corresponding variables are significantly correlated with depression (*P* value < 0.05); adjusted odds ratio showed the nature of the relationship.
